# Supplementary material for: Evolution of Complex Modular Biological Networks
Source: PLoS Comput Biol. 2008 Feb 8;4(2):e23. doi: 10.1371/journal.pcbi.0040023 (PMC2233666; doi:10.1371/journal.pcbi.0040023)
Supplement: Supplementary file 1 [file pcbi.0040023.sg001.pdf]

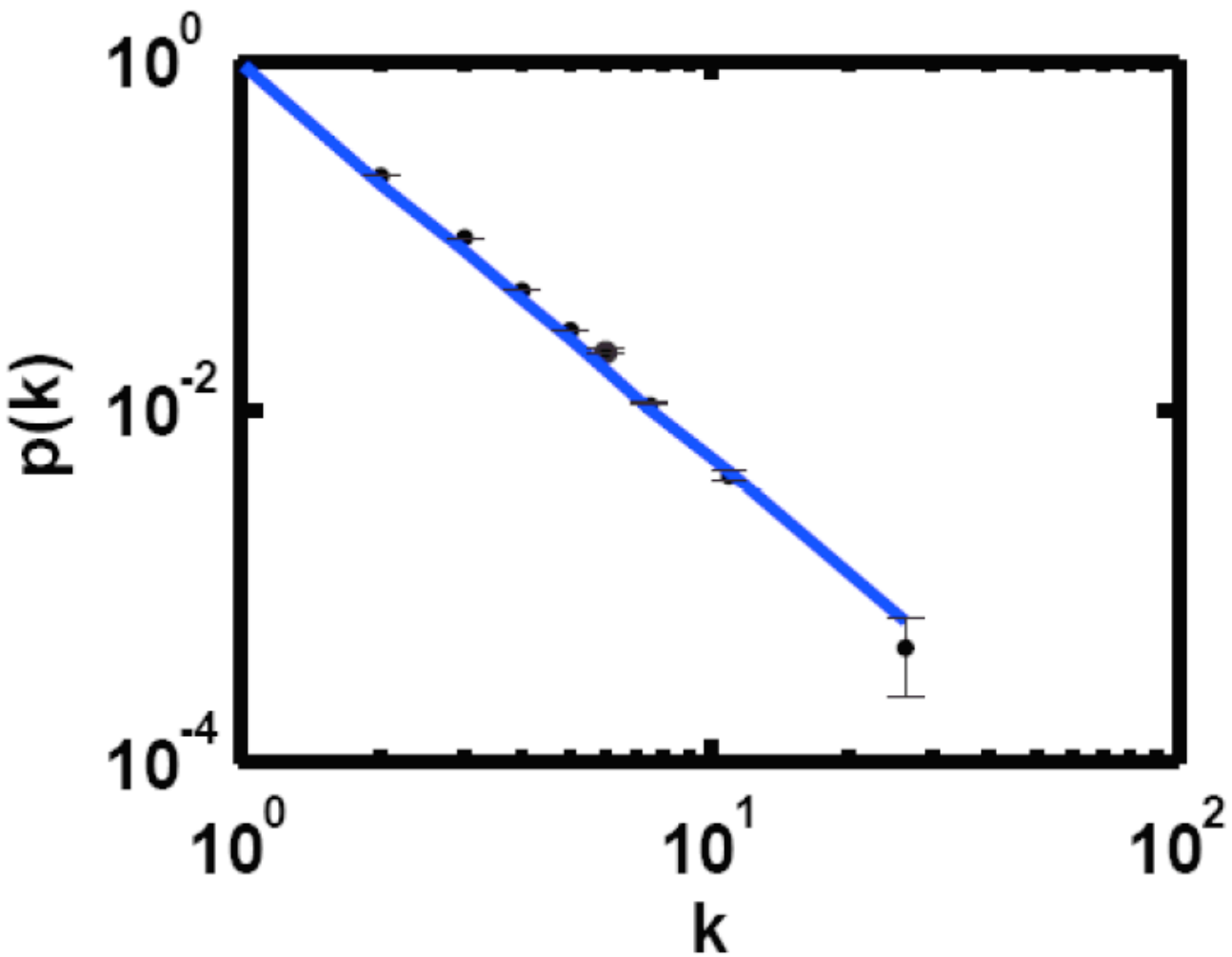

Figure S1. Distribution of molecules in reactions

Probability distribution *p(k)* that a molecule participates in *k* reactions, compiled from 80 runs to depth 1,000 in a dynamic environment. The distribution is fit to a power law $p(k) \sim k^{-\lambda}$, with $\lambda \approx 2.23$ (r^2=0.88). Error bars are standard error. Variable bin sizes are determined by the threshold binning method [37], with a minimum of *T*=100 points per bin.
