# Supplementary material for: Evolution of Complex Modular Biological Networks
Source: PLoS Comput Biol. 2008 Feb 8;4(2):e23. doi: 10.1371/journal.pcbi.0040023 (PMC2233666; doi:10.1371/journal.pcbi.0040023)
Supplement: Supplementary file 2 [file pcbi.0040023.sg002.pdf]

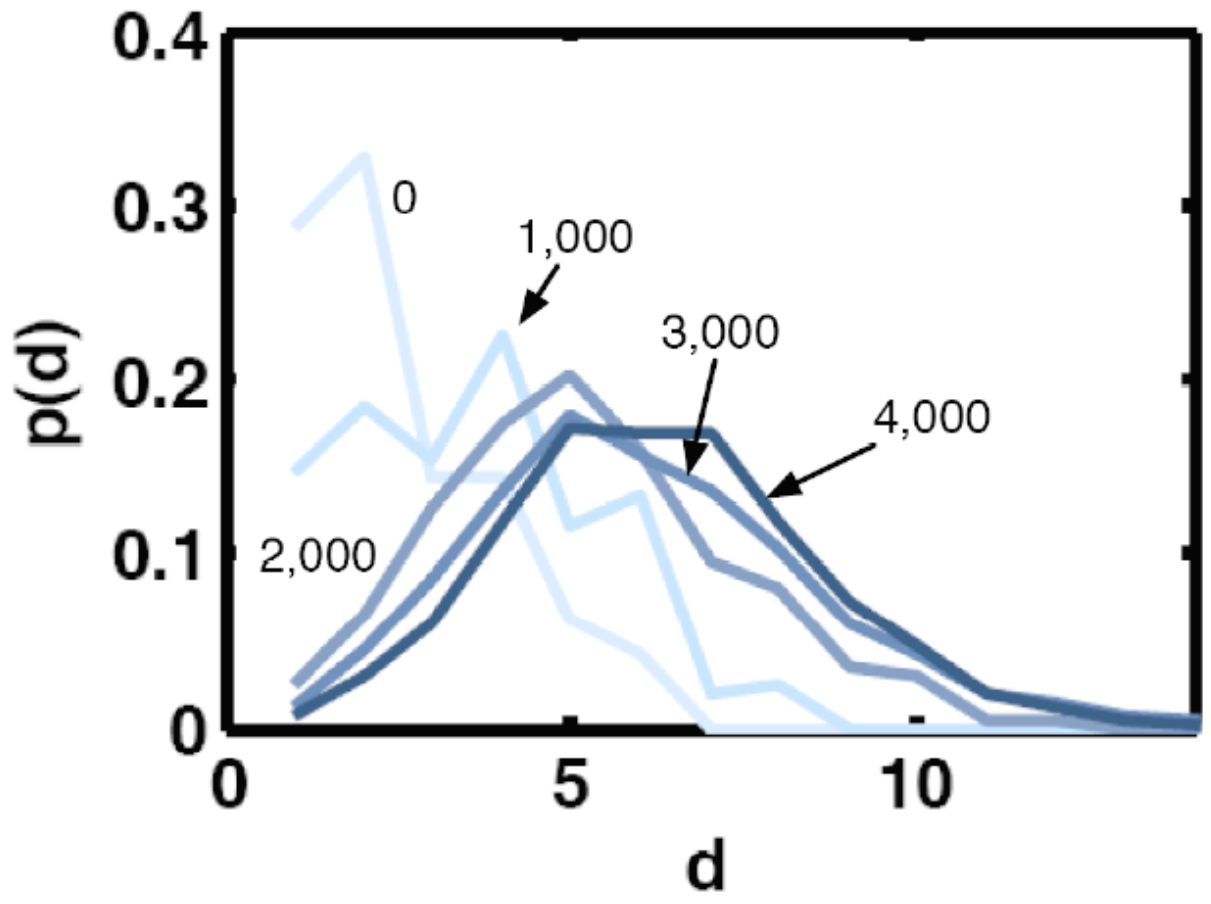

Figure S2. Evolution of path length distribution

Evolution of the distribution *p(d)*, the probability to find two nodes in the network that are a distance *d* apart, for every 1,000th network on the line of descent, for a network evolved in a dynamic environment.
