# Supplementary material for: Evolution of Complex Modular Biological Networks
Source: PLoS Comput Biol. 2008 Feb 8;4(2):e23. doi: 10.1371/journal.pcbi.0040023 (PMC2233666; doi:10.1371/journal.pcbi.0040023)
Supplement: Supplementary file 3 [file pcbi.0040023.sg003.pdf]

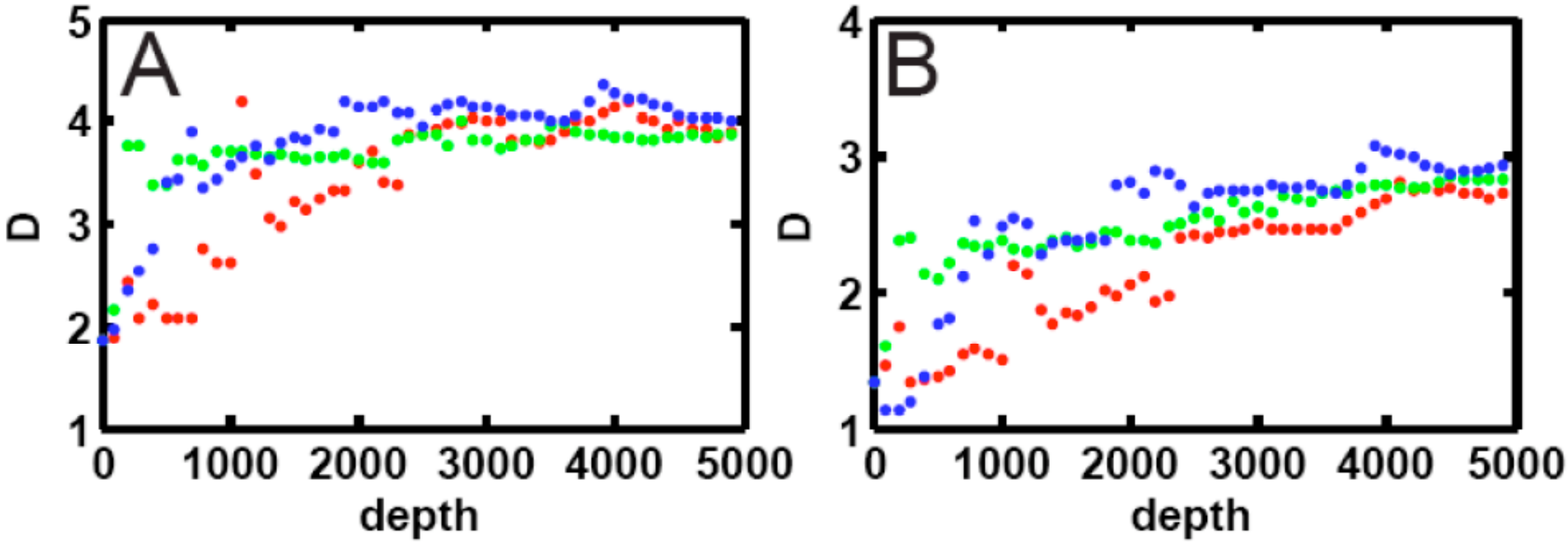

Figure S3. Average path length D on the line of decent

Mean path length *D* (see Methods) for a network with **(A)** metabolic, and **(B)** protein-protein annotation, in three different environments, for the network evolution shown in Fig. 2. Green: static, blue: quasi-static, and red: dynamic environment.
