# Supplementary material for: Evolution of Complex Modular Biological Networks
Source: PLoS Comput Biol. 2008 Feb 8;4(2):e23. doi: 10.1371/journal.pcbi.0040023 (PMC2233666; doi:10.1371/journal.pcbi.0040023)
Supplement: Supplementary file 4 [file pcbi.0040023.sg004.pdf]

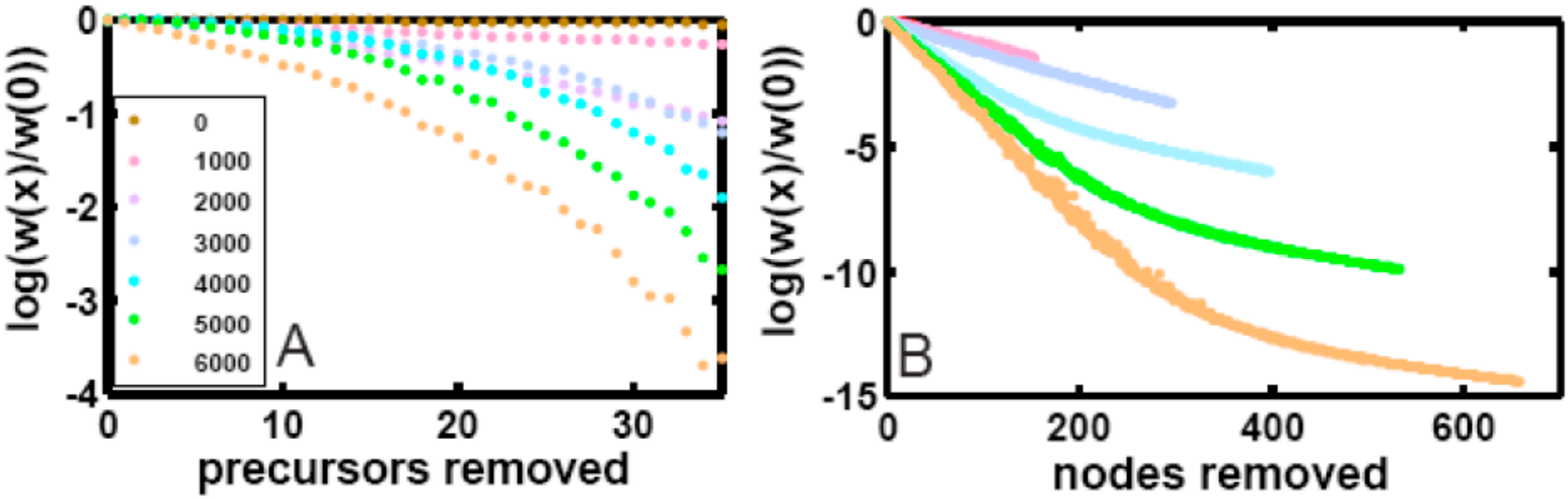

Figure S4. Robustness of fitness under precursor and gene removal

Decrease of normalized log fitness with increasing **(A)** precursor removal, **(B)** node removal, as a function of the position on the line of descent (colors in inset of panel A). Depth 0: ancestor.
