# Supplementary material for: Evolution of Complex Modular Biological Networks
Source: PLoS Comput Biol. 2008 Feb 8;4(2):e23. doi: 10.1371/journal.pcbi.0040023 (PMC2233666; doi:10.1371/journal.pcbi.0040023)

Figure S5. Modularity analysis for static and quasi-static environments

Analysis of the separation of pairs of genes from networks evolved in an **(A)** static and **(B)** quasi-static environment, as in Fig. 8A. Red line: synthetic lethal pairs, green: dosage rescue pairs, black: random pairs and grey: relative size of largest connected component.

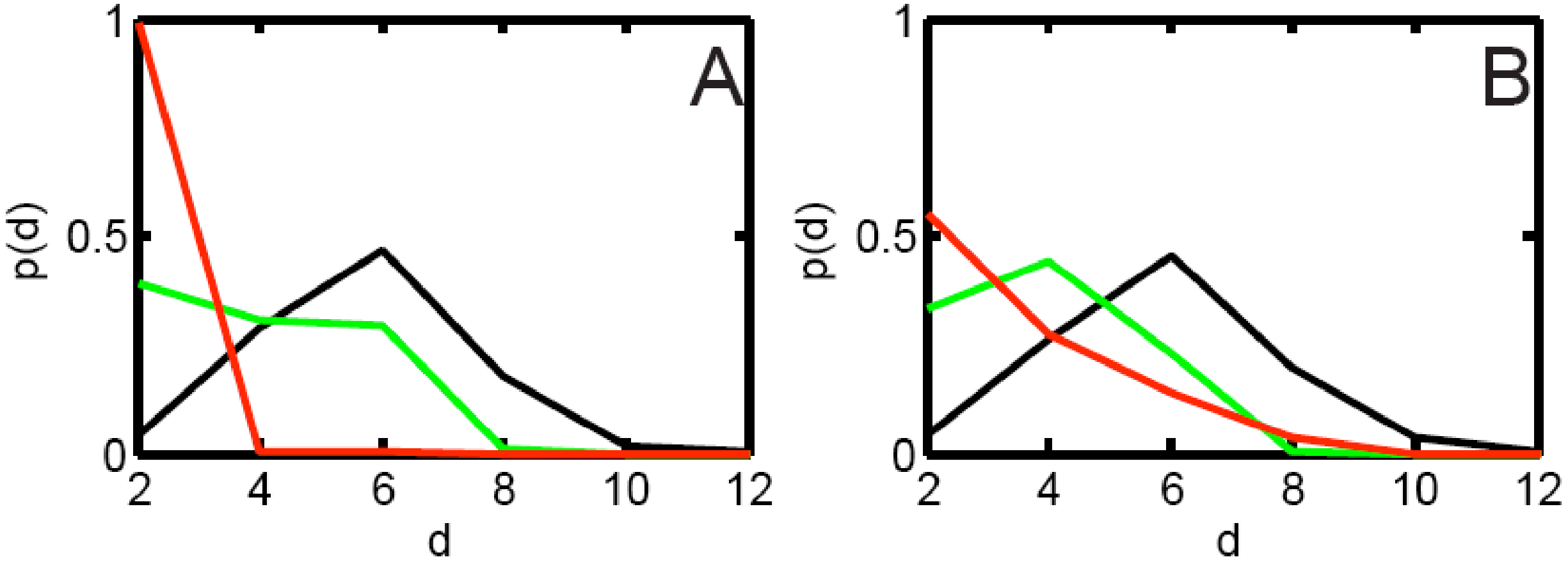

Supplement: Supplementary file 5 [file pcbi.0040023.sg005.pdf]
