# Supplementary material for: Evolution of Complex Modular Biological Networks
Source: PLoS Comput Biol. 2008 Feb 8;4(2):e23. doi: 10.1371/journal.pcbi.0040023 (PMC2233666; doi:10.1371/journal.pcbi.0040023)
Supplement: Supplementary file 6 [file pcbi.0040023.sg006.pdf]

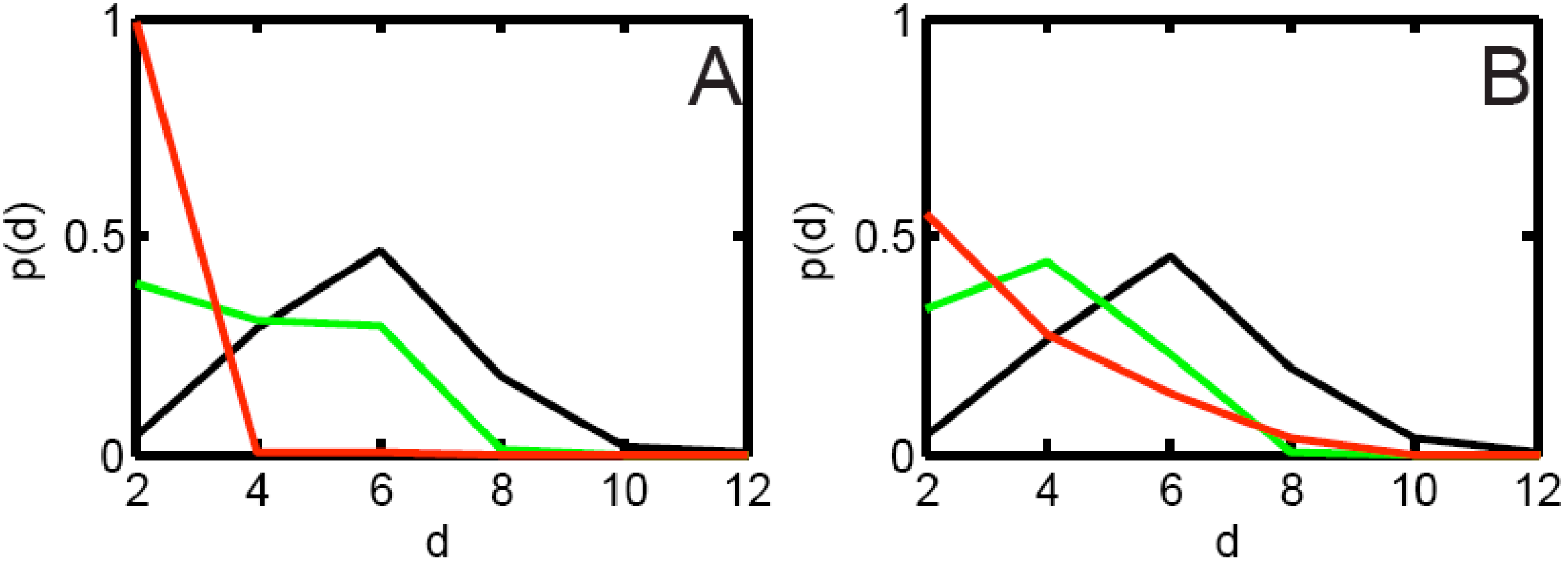

Figure S6. Distance distribution of pairs of genes

Distance distribution of pairs on a network evolved in (**A**) static and (**B**) quasi-static environment. Red: synthetic lethal pairs, green: knockdown suppressor pairs, black: random pairs.
