# Supplementary material for: Evolution of Complex Modular Biological Networks
Source: PLoS Comput Biol. 2008 Feb 8;4(2):e23. doi: 10.1371/journal.pcbi.0040023 (PMC2233666; doi:10.1371/journal.pcbi.0040023)
Supplement: Supplementary file 7 [file pcbi.0040023.sg007.pdf]

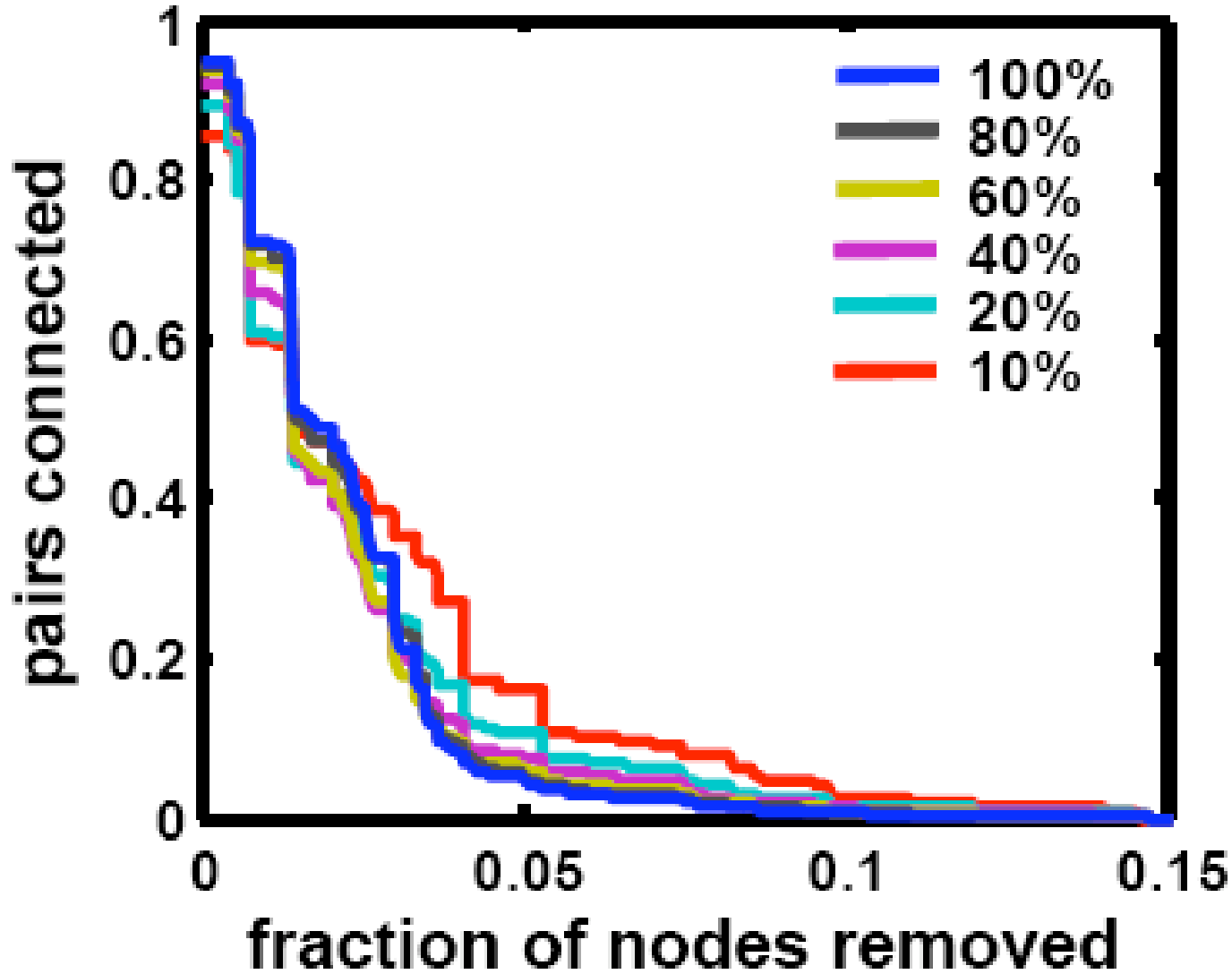

Figure S7. Robustness of decay of knockdown suppressor pairs

Fraction of knockdown suppressor pairs separated upon removing nodes with high BC using all (100%, weakest criterion) or fewer (only the top 10-80%) of suppressor pairs. The top 95% of pairs were used for Figures 8A and S5. See legend for colors and thresholds.
