# Supplementary material for: Evolution of Complex Modular Biological Networks
Source: PLoS Comput Biol. 2008 Feb 8;4(2):e23. doi: 10.1371/journal.pcbi.0040023 (PMC2233666; doi:10.1371/journal.pcbi.0040023)
Supplement: Supplementary file 8 [file pcbi.0040023.sg008.pdf]

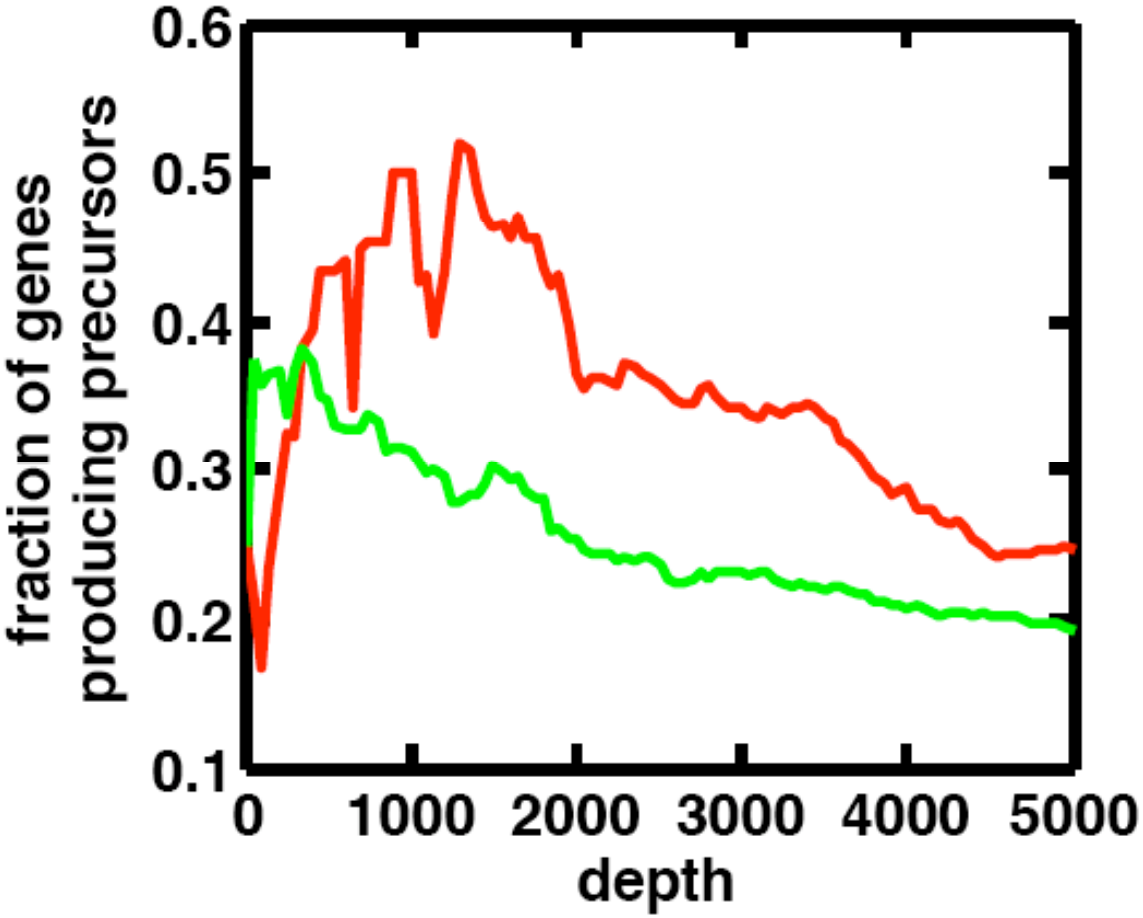


**Figure S8. Fraction of genes producing precursors**
Fraction of genes involved in the production of one of the 53 precursor molecules for the network evolved in a dynamic environment (red) vs. a static environment (green line).
