# Supplementary material for: Evolution of Complex Modular Biological Networks
Source: PLoS Comput Biol. 2008 Feb 8;4(2):e23. doi: 10.1371/journal.pcbi.0040023 (PMC2233666; doi:10.1371/journal.pcbi.0040023)
Supplement: Supplementary file 9 [file pcbi.0040023.st001.pdf]

Table S1. Organization of a 72 bp gene

Base pair	Parameter
0-3	Start
4-7	Expression level (converted to real number between 0 and 1)
8-11	Type of protein, obtained by taking the base 4 modulus of the sequence. 00=import, 01=export, 10=reaction, 11=reaction
12-23	Specificity of protein
24-35	Affinity domain 1
36-47	Affinity domain 2
48-59	Affinity domain 3
60-71	Affinity domain 4
